# Rare submucosal abscess following endoscopic mucosal resection for rectal polyps: a case report and literature review

**DOI:** 10.3389/fmed.2026.1829122

**Published:** 2026-05-14

**Authors:** Guo-Li Cao, Ze-Ming Chen, Xiao-Dong Li, Xi-Qiu Yu

**Affiliations:** Department of Gastroenterology, Shenzhen Luohu People's Hospital, The Third Affiliated Hospital of Shenzhen University, Shenzhen, China

**Keywords:** case report, endoscopic mucosal resection, phlegmon, Rectal abscess, rectal polyps

## Abstract

Colonoscopy combined with polypectomy is a well-established strategy for the prevention of colorectal cancer, and endoscopic mucosal resection (EMR) serves as one of the primary therapeutic modalities for colorectal neoplastic lesions. While EMR is generally safe, severe complications may occasionally occur. Herein, we report a rare case of rectal submucosal abscess in a 74-year-old female patient who underwent EMR for rectal polyps. Initial endoscopic fenestration and drainage of the abscess failed to achieve a satisfactory therapeutic effect, and the patient ultimately underwent sigmoidostomy for further management. This case highlights the necessity of considering phlegmon or abscess formation in the differential diagnosis of patients presenting with unexplained acute abdomen after EMR for rectal lesions, especially in those with compromised immunity. Timely identification and intervention are crucial to avoid adverse clinical outcomes.

## Introduction

Endoscopic polypectomy is an effective strategy for preventing the development of colorectal cancer (1), and EMR is a routine and well-recognized method for the treatment of colorectal neoplastic lesions (2). Bleeding, perforation, and post-polypectomy syndrome (PPS) are the main complications of EMR, and most of these cases can be successfully managed without surgical intervention (2, 3). However, rare but life-threatening infectious complications such as submucosal abscess following EMR are extremely rare and easily overlooked in clinical practice. Several case studies have reported rare complications such as abscess formation and phlegmonous enteritis in patients undergoing endoscopic submucosal dissection (ESD) (4, 5). To the best of our knowledge, there are no reports in the literature of colonic wall abscess induced by EMR. Given the low incidence and potentially severe clinical course, early recognition and proper management of this rare complication are critical for gastroenterologists. Therefore, we report the first case of submucosal abscess following EMR for a 6 mm rectal polyp, with comprehensive clinical data, imaging features, microbiology results, and a systematic literature review. This case aims to improve awareness of this rare adverse event and optimize clinical decision-making for high-risk patients.

## Case presentation

**Figure 1:**
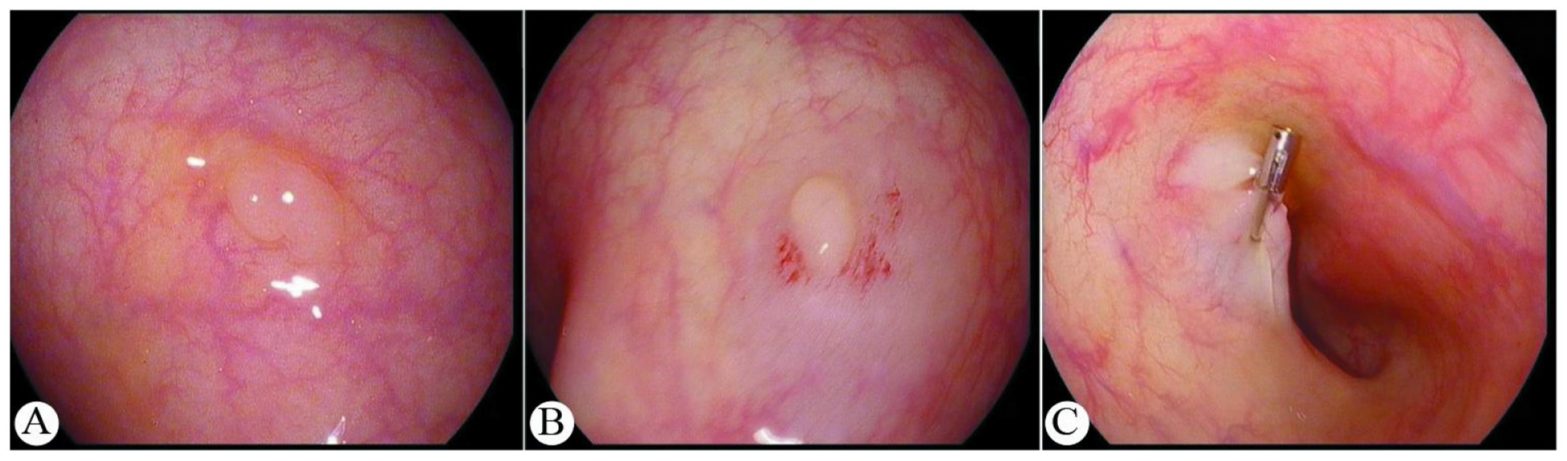
EMR for a rectal polyp. **(A)** Endoscopic image of the rectal polyp. **(B)** Submucosal injection of a saline-epinephrine mixture into the submucosa to elevate the polyp. **(C)** Closure of the mucosal defect with a titanium clip following EMR.

A 74-year-old female patient was admitted to the hospital for endoscopic polypectomy following the diagnosis of rectal polyps during a routine check-up. Her medical history included incomplete intestinal obstruction 1 year prior and a hysterectomy for uterine prolapse 5 years prior. Routine laboratory tests upon admission revealed no obvious abnormalities. The patient received bowel preparation with 2 L of polyethylene glycol solution, followed by colonoscopy under intravenous anesthesia. The quality of bowel preparation was rated excellent according to the Boston Bowel Preparation Scale (6). During colonoscopy, a 6 mm sessile polyp was identified in the rectum. The polyp was resected via EMR following submucosal injection of diluted epinephrine, and the resection site was closed with titanium clips after the procedure (Figure 1). The pathological examination results revealed tubular adenoma. The operation was performed by an experienced endoscopist, lasting approximately 30 min with no intraoperative complications observed. The patient received fasting and fluid replacement therapy postoperatively and reported no specific discomfort on the day of the procedure.

On the morning of first postoperative day, the patient developed chills and fever with a maximum body temperature of 39.3 °C, accompanied by lower abdominal discomfort. Physical examination revealed mild tenderness in the lower abdomen without rebound tenderness. Blood tests showed a white blood cell (WBC) count of 12.62 × 10^9^/L and a neutrophil percentage of 89.5%, with no abnormal levels of high-sensitivity C-reactive protein (CRP) or procalcitonin (PCT). Based on our clinical experience, PPS was suspected, and the patient was administered cefuroxime for anti-infection treatment. On the third postoperative day, her body temperature returned to normal and lower abdominal discomfort was relieved. Despite medical advice for further hospitalization and observation, the patient insisted on discharge and was prescribed oral cefuroxime for continued treatment.

**Figure 2:**
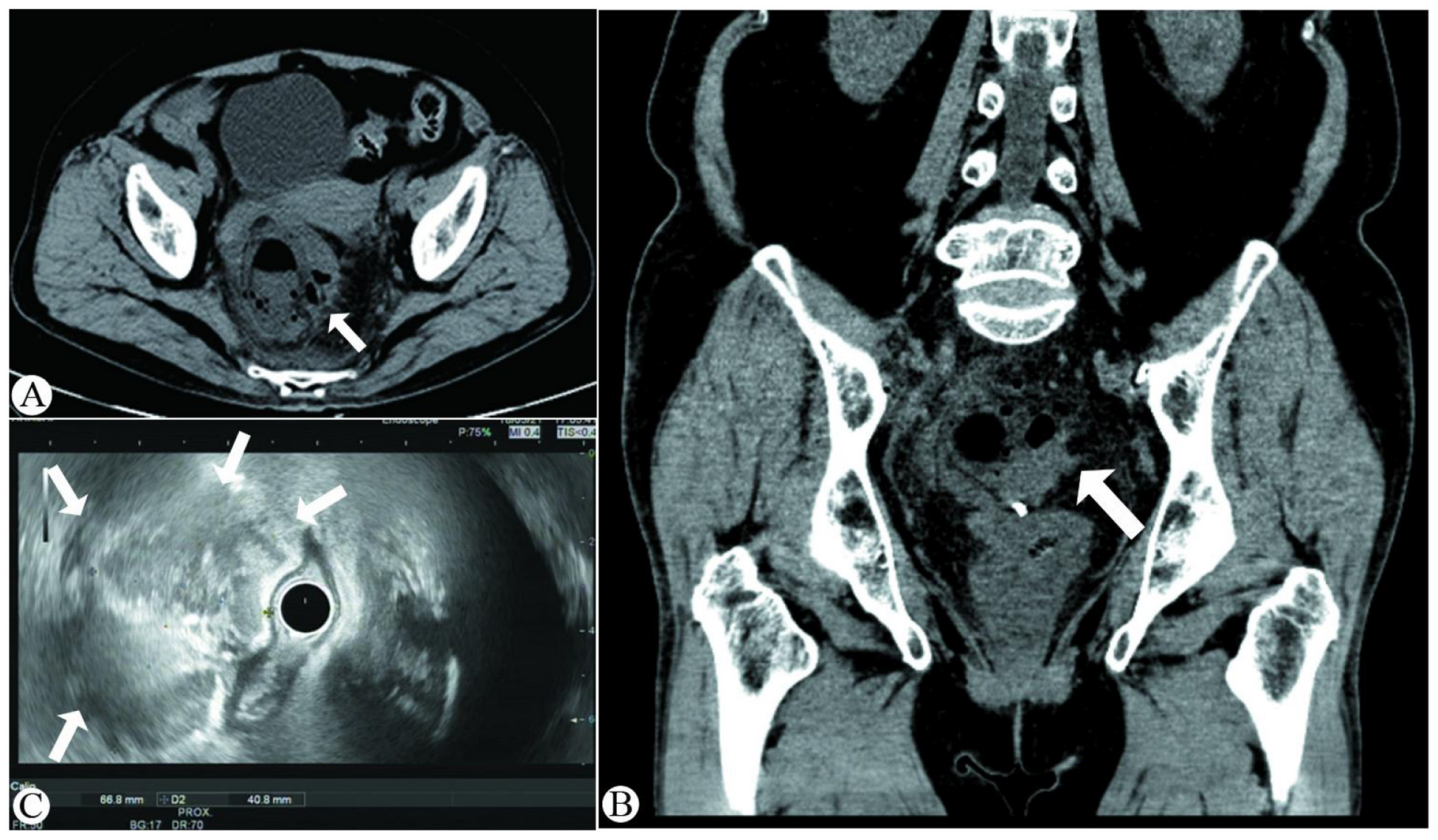
Post-EMR imaging findings of the patients. **(A, B)** CT scans demonstrating rectal wall thickening, associated with intramural gas and blurred perirectal fat spaces (white arrow). **(C)** EUS (7.5 MHz) revealing a hypoechoic lesion arising from the second layer of the rectal wall (white arrow).

**Figure 3:**
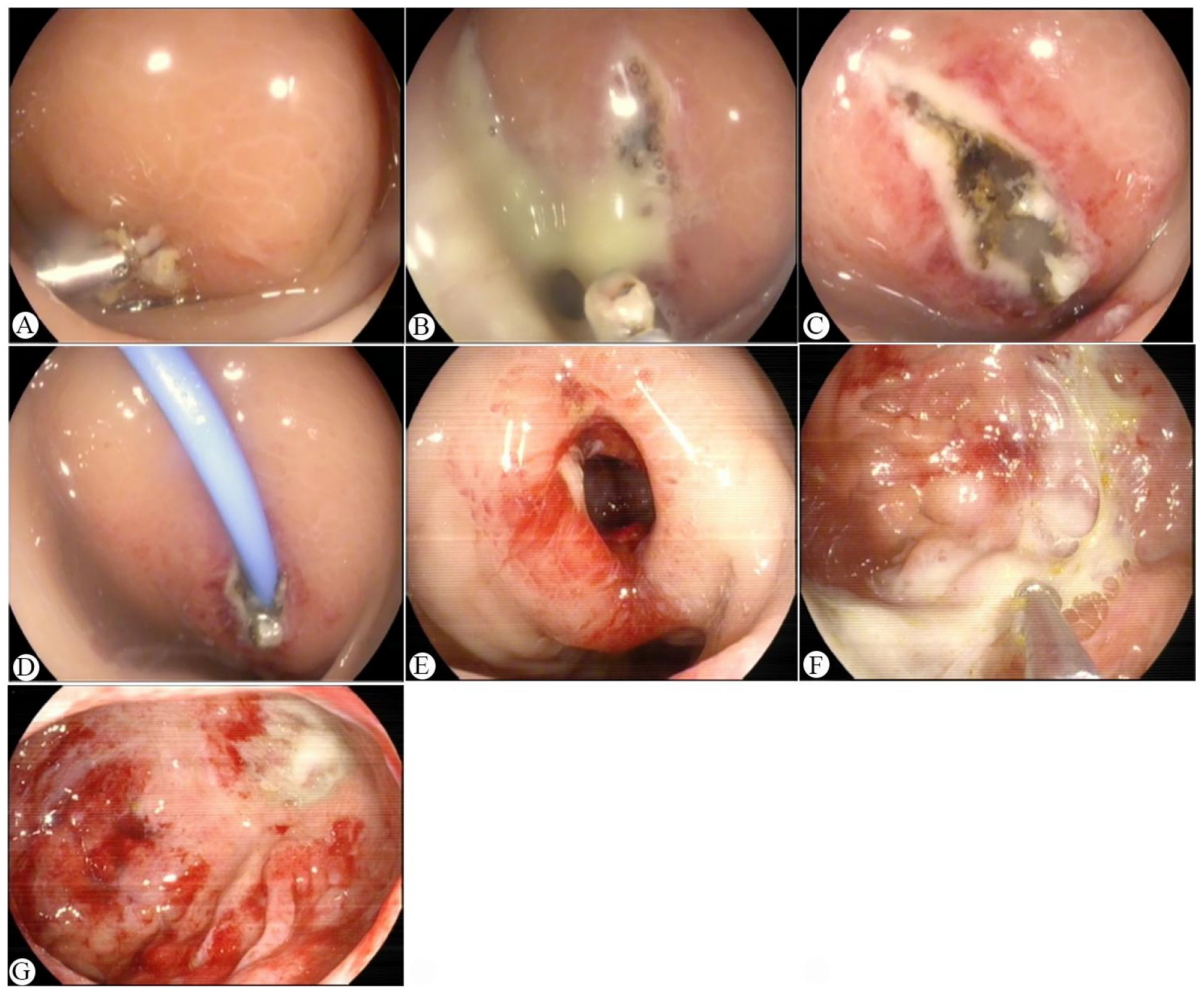
Endoscopic fenestration and drainage for rectal submucosal abscess. **(A)** Submucosal bulging with purulent exudate noted at the rectal post-EMR site; **(B)** Titanium clip removal with subsequent drainage of a large volume of pus; **(C)** Endoscopic fenestration performed with a snare; **(D)** Placement of a drainage catheter; **(E)** Repeat colonoscopy at 5 days post-abscess drainage showing the drainage orifice; **(F)** Granulation tissue and residual pus visualized upon entry into the abscess cavity; **(G)** Morphological changes of the abscess cavity after irrigation.

The patient was readmitted 4 days after discharge (7th postoperative day), reporting persistent intermittent fever, aggravated distending pain in the lower abdomen, and anal distension over the previous 3 days. Her body temperature was normal on readmission. Physical examination showed tenderness in the lower abdomen without rebound tenderness. Repeat laboratory tests revealed a WBC count of 13.9 × 10^9^/L, a neutrophil percentage of 88.0%, and a CRP level of 89 mg/L, with normal serum PCT. Abdominal computed tomography (CT) showed intestinal dilatation and bowel wall thickening in the rectal region, accompanied by blurred perienteric fat spaces, suggesting postoperative infection and abscess formation (Figures 2A, B). An emergency colonoscopy was subsequently performed, revealing post-EMR changes with residual titanium clips 10 cm from the anal verge; the area was bulging and swollen with purulent secretions on the surface (Figure 3A). Further endoscopic ultrasonography (EUS) demonstrated a large hypoechoic lesion with an ill-defined boundary, approximately 5 cm in diameter, originating from the submucosa (Figure 2C). A diagnosis of rectal submucosal abscess following EMR for rectal polyps was thus confirmed. Subsequently, endoscopic fenestration was performed for the patient. Titanium clips were first removed with a foreign body forceps, and the fenestration was further enlarged with a snare to allow the drainage of a large amount of pus. Meanwhile, the abscess cavity was irrigated with metronidazole, and a 7.5 Fr endoscopic nasobiliary drainage catheter was placed for adequate pus drainage (Figures 3B–D). Postoperatively, the patient was managed with fasting, anti-infection treatment with cefoperazone sodium, parenteral nutritional support, and daily irrigation of the abscess cavity.

On the first day after drainage, the patient's temperature remained stable without recurrence of fever, and abdominal pain was significantly alleviated. Repeat complete blood count showed a WBC count of 8.9 × 10^9^/L with a neutrophil percentage of 80.3% and a CRP level of 73 mg/L. The pus culture identified Escherichia coli. Repeat colonoscopy 5 days after drainage revealed no substantial evidence of healing within the abscess cavity (Figures 3E, G). To optimize infection control and promote abscess healing, the patient underwent sigmoidostomy after consultation with the general surgery team.

**Figure 4:**
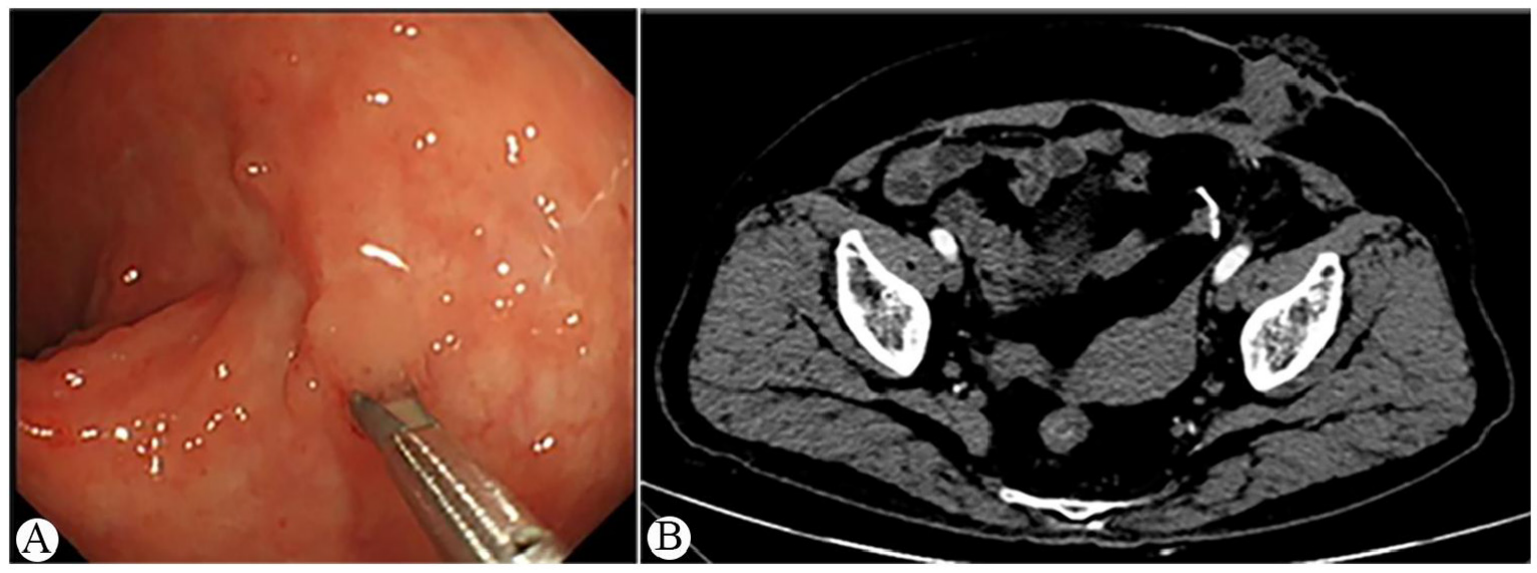
Final follow-up colonoscopic and CT findings. **(A)** Repeat colonoscopy at 115 days after polypectomy showing well-healed intestinal mucosa with a residual titanium clip; **(B)** Follow-up CT scan confirming complete resolution of the previously identified rectal wall abscess.

The patient had an uneventful postoperative recovery and was discharged on the 12th postoperative day. A follow-up colonoscopy at 3 months postoperatively showed complete mucosal healing of the rectal wall, and enhanced abdominal CT confirmed the complete resolution of the previously identified rectal submucosal abscess (Figure 4). The patient subsequently underwent successful sigmoidostomy closure with no postoperative complications and remained in good condition at the 1-year follow-up.

## Discussion

EMR is currently the first-line treatment for large sessile flat colorectal lesions without submucosal invasion and is generally regarded as a relatively safe therapeutic option for benign colorectal lesions (7). The main unexpected complication during colonoscopic treatment is bleeding, followed by the relatively rare complications of perforation and PPS (8–10); postoperative infection occurs infrequently (11). EMR is currently the first-line treatment for large sessile flat colorectal lesions without submucosal invasion and is generally regarded as a relatively safe therapeutic option for benign colorectal lesions (7). The main unexpected complication during colonoscopic treatment is bleeding, followed by the relatively rare complications of perforation and PPS (8–10); postoperative infection occurs infrequently (11). Submucosal injection of a lifting solution can reduce the risk of perforation. In addition, using clips for hemostasis is an effective method for managing post-polypectomy bleeding and preventing postoperative perforation (12). A prospective study reported that the incidence of transient bacteremia associated with EMR or ESD for colonic lesions is approximately 2.5%, with no patients presenting with infection-related symptoms or signs (13). A prospective study reported that the incidence of transient bacteremia associated with EMR or ESD for colonic lesions is approximately 2.5%, with no patients presenting with infection-related symptoms or signs (13). Phlegmon is a diffuse suppurative inflammatory process primarily involving the submucosa, which may spread to the serosa and present as peritonitis. Colorectal abscess, a localized manifestation of phlegmonous enteritis, is characterized by localized pus accumulation accompanied by tissue destruction (4, 14). It is a rare but severe complication after polypectomy. To the best of our knowledge, this is the first reported case of intramural abscess developing after rectal EMR.

To identify existing literature on phlegmon following colonoscopy or related treatments, we conducted a comprehensive search across multiple databases, including Google Scholar, Medline, Web of Science, and Embase, using the keywords: phlegmon, phlegmonous enteritis, phlegmonous colitis, abscess, endoscopic mucosal resection, endoscopic submucosal dissection, polypectomy, and endoscopic treatment. Current literature only documents one case of phlegmon after ESD and one case after cold snare polypectomy (4, 15). In addition, two previous cases of gastric submucosal abscess after EMR have been recorded (16, 17), and no case reports have detailed submucosal abscess as a complication of EMR for colorectal polyps. Furthermore, the literature has documented five additional cases of phlegmonous gastritis after ESD and polypectomy (5, 18–21). Given the common procedural interventions in these cases, which may indicate a shared pathogenesis, we summarized these 10 cases and briefly presented them in Table 1. Notably, several case reports have described the formation of intraperitoneal and retroperitoneal abscesses after polypectomy (11, 22–24); however, the pathophysiological mechanism of such cases is usually attributed to perforation or unrecognized microperforation, which differs from the cases mentioned above and thus they were not included in the current discussion.

**Table 1:** Reported cases of phlegmonous enteritis or perigastric abscess after endoscopic treatment in the literature.

Study	Year	Age (yr)	Sex	Type	Proce-dure	Underlying medical condition	Prophy-lactic antibiotics	Symptom	Time of symptom onset post-operative	Diagnostic method	Pathogens	Treatment method	Outcome
LIFTON et al. (21)	1982	70	Female	Phlegmonous gastritis	HSP	None	No	Adominal pain, fever, nausea, vomiting	1 day	Barium X-ray, EUS	Strep	Antibiotics, gastrectomy	Cure
Lee et al. (17)	2005	68	Female	Phlegmonous gastritis	EMR	None	No	Abdominal pain, fever	8 h	CT	E. faecalis	Antibiotics, total gastrectomy	Cure
Ajibe et al. (20)	2008	74	Male	Phlegmonous gastritis	ESD	DM, CKD	No	Abdominal pain, fever	5 h	CT, EUS	C. freundii, ECL, Strep	Antibiotics, total gastrectomy	Cure
Dohi et al. (19)	2014	63	Female	Gastric wall abscess	ESD	None	No	Abdominal pain	5 days	CT, MRI	Not described	Antibiotics, endoscopic drainage	Cure
Matsuura et al. (18)	2018	76	Female	Phlegmonous gastritis	ESD	DM, MDS	No	Fever	1 day	CT	KP, PA, CAL	Antibiotics	Cure
Yu et al. (5)	2018	72	Female	Gastric wall abscess	ESD	HTN	No	None	10 weeks	CT	Not described	Subtotal gastrectomy	Cure
Li et al. (16)	2024	68	Male	Gastric wall abscess	EMR	Dermatomyositis	No	Abdominal pain	3 days	CT, EUS	ABA	Antibiotics	Cure
Tian et al. (4)	2024	66	Female	Phlegmonous colitis	ESD	HT	No	Abdominal pain, fever	4 h	CT	E. coli	Antibiotics, right hemicolectomy	Cure
Kimura et al. (15)	2024	78	Male	Phlegmonous colitis	CSP	CMML, MI, DM, CKD, and ASO	No	Abdominal pain, fever	8 days	CT	detected	Antibiotics	Cure
Present case	2026	74	Female	Colonic wall abscess	EMR	Large bowel obstruction, Uterine prolapse	No	Abdominal pain, fever	1 day	CT, EUS	E. coli	Antibiotics, endoscopic drainage, sigmoidostomy	Cure

ESD, endoscopic submucosal dissection; EMR, endoscopic mucosal resection; CSP, cold snare polypectomy; HSP, hot snare polypectomy; DM, diabetes mellitus; CKD, chronic kidney disease; HTN, hypertension; MDS, myelodysplastic syndrome; HT, hashimoto's thyroiditis; CMML, chronic myelomonocytic leukemia; MI, myocardial infarction; ASO, arteriosclerosis obliterans; CT, computed tomography; EUS, endoscopic ultrasonography; MRI, magnetic resonance imaging; E. faecalis, Enterococcus faecalis; C. freundii, Citrobacter freundii; ECL, Enterobacter cloacae; Strep, Streptococcus; KP, Klebsiella pneumoniae; PA, Pseudomonas aeruginosa; CAL, Candida albicans; ABA, Acinetobacter baumannii; E. coli, Escherichia coli.

Immunocompromised conditions, including advanced age and underlying chronic diseases, often predispose patients to post-endoscopic infection (5, 25). Among the 10 cases integrated in this study, all patients were elderly (median age 71 years, range 63–78 years), and seven (70%) had a history of underlying diseases. This highlights the need for special attention to the occurrence of postoperative infection in elderly patients with comorbidities. In addition, intestinal bacterial translocation is the direct source of infection (8). During EMR, factors such as large polyp size or prolonged electrocautery may cause necrosis of the rectal submucosal tissue, creating conditions conducive to bacterial colonization (4, 15). Intestinal bacteria invade the submucosa through mucosal lacerations, leading to infection and abscess formation. Another potential cause of infection in patients is that, on the basis of impaired immune function, bacteria-contaminated injection needles may introduce bacteria into the submucosa during EMR, which then gradually spread in the colonic wall and eventually colonize the submucosal tissue, forming a submucosal abscess (16, 26). Meanwhile, the formation of a submucosal hematoma creates nutritional conditions for bacterial reproduction, thereby exacerbating infection and increasing the risk of abscess formation (27). In this case, the patient underwent EMR for rectal polyps, and the resulting mucosal defect was closed with titanium clips. We speculate that the formation of the rectal submucosal abscess was mainly due to bacteria entering the submucosa through the injection needle, and the patient's impaired immune function may have exacerbated this process. Microbiological analysis of the abscess pus yielded Escherichia coli, a ubiquitous commensal member of physiological human intestinal flora. This microbiological evidence directly supports our mechanistic hypothesis: during the submucosal injection step of EMR, the injection needle translocated endogenous intestinal bacteria into the submucosal compartment. Subsequent bacterial colonization and proliferation in the submucosa evoked sustained inflammatory responses, which ultimately culminated in the formation of the submucosal abscess. An alternative explanation, however, is that bacteria entered through the mucosal defect after surgery, leading to subsequent abscess formation.

Gastrointestinal phlegmon typically presents with acute abdominal pain and fever (4). According to our integrated data, 80% of patients with phlegmon after endoscopic treatment presented with these symptoms, and only one patient was completely asymptomatic. This condition shares the same clinical features with other diseases causing acute abdominal pain, suggesting that phlegmon or abscess formation should be considered in patients with acute abdominal pain and elevated body temperature after endoscopic treatment, with timely diagnostic evaluation to reduce adverse outcomes.

CT scanning can display the location, size, and scope of abscesses and enable the early detection of complications such as abscess and perforation (28). Magnetic resonance imaging (MRI) exhibits higher sensitivity than CT in detecting intestinal abscesses (29). EUS can clearly show the structure of the submucosa, determine the location and size of submucosal lesions and their relationship with surrounding tissues, and guide puncture and drainage, thus playing an important role in the diagnosis and treatment of submucosal abscesses (28, 30). Bacterial culture of pus can ultimately confirm the diagnosis of abscess and guide the use of antibiotics. The most common endogenous pathogens causing intestinal abscesses include Escherichia coli, Klebsiella, other Enterobacteriaceae, and Enterococci (8). The administration of prophylactic antibiotics for colonic lesions after EMR or ESD remains a controversial topic. Given the low incidence of postoperative infection and abscess formation, routine prophylactic use of antibiotics is not recommended (9, 13). A meta-analysis found that prophylactic use of cefixime and cefuroxime can reduce the infection rate and abscess formation to as low as 0% and 0.76%, respectively. Individualized management should be determined based on clinical conditions, and postoperative prophylactic antibiotics may be appropriate for immunocompromised individuals at high risk of abscess formation.

The treatment of submucosal abscess includes antibiotic therapy, puncture and drainage, and surgical intervention. Antibiotics are particularly suitable for patients with early extensive phlegmon and small-sized abscesses (15, 18, 19, 31). The main treatment for colorectal abscess is incision and drainage (29, 32). Endoscopic drainage is a highly effective method for the treatment of submucosal abscess, including EUS-guided puncture and drainage and endoscopic incision and drainage. EUS-guided puncture allows precise puncture of the abscess cavity, facilitating drainage tube placement and promoting pus discharge (28, 30), while endoscopic incision and drainage is particularly suitable for superficial abscesses, with pus drained by incising the mucosa endoscopically (25, 32). Fenestration under electronic colonoscopy for the treatment of colorectal abscess has the advantages of simple operation, safety and reliability (32). However, early surgical intervention is required for patients with resistance to antibiotic treatment, large abscesses, or complicated perforation (33). Acute anorectal abscess should be treated with timely incision and drainage (29). Among the included studies, three patients were successfully cured with conservative anti-infection treatment, one patient with endoscopic drainage combined with anti-infection treatment, and the other six patients ultimately required surgical intervention. In this case, the patient was infected with Escherichia coli developed a large rectal submucosal abscess; she initially received antibiotic treatment and endoscopic abscess drainage. However, considering the patient's impaired immune function and inadequate healing of the abscess cavity, there was a risk of persistent lesions and inflammatory spread, and thus surgical intervention was immediately selected as the definitive treatment for the rectal submucosal abscess.

## Conclusion

This report presents the first documented case of a submucosal abscess following EMR of a rectal polyp. This case underscores the necessity of considering the possibility of abscess formation in the differential diagnosis of unexplained acute abdominal pain following EMR of rectal lesions, particularly in immunocompromised patients at high risk of phlegmon and abscess formation. This case not only enriches the existing literature on EMR-related complications but also underscores the importance of timely recognition and targeted intervention for rectal submucosal abscess to prevent adverse clinical outcomes.

## Data Availability

Publicly available datasets were analyzed in this study. The data presented in this study are derived from the clinical records of a single patient and are not publicly available due to privacy and ethical restrictions. All relevant clinical information is summarized within the manuscript.
